# Development of a Mental Health Strategy in an Acute Trust – A New Role for Psychiatrists

**DOI:** 10.1192/bjo.2025.10347

**Published:** 2025-06-20

**Authors:** Samuel Dearman, Roger Cable

**Affiliations:** NCIC, Carlisle, United Kingdom

## Abstract

**Aims:** The aim was to develop a strategy for mental health and learning disabilities in an acute and community trust in North Cumbria, in response to the CQC report “Assessment of mental health services in acute trusts programme” published in 2020. The strategy also needed to align with the trust vision, values and objectives while developing a clear but simple overview of what is required to improve mental health services within the trust for people with a wide range of mental health difficulties and learning disabilities. We also describe novel and innovative roles for psychiatrists as a new area of professional practice.

**Methods:** The trust appointed strategic and clinical leads, both consultant psychiatrists supported by a senior manager. We reviewed the latest government documents, NHS guidelines and college reports in relation to mental health priorities within an acute trust, reviewed the mental health service delivery requirements as set out by the CQC both nationally and by analysing the local CQC report. Existing services within the trust and partner organisations such as social care, other NHS trusts, primary care and the ICB were consulted.

**Results:** The strategy was developed and focused on 5 tactical arms:

Culture, kindness, inclusion and understanding.

Shared patients, partnership and policy.

NCIC innovations.

Integrated governance.

Organisation of roles.

Each arm has a concise description of what needs to be done to achieve our strategic aims with a set of key performance indicators to evaluate the trust’s performance in achieving the CQC requirements. The strategy has been underpinned by developing an oversight group to understand the strengths and areas needing improvement, thus informing the appropriate development of services and resource allocation. The strategy has been approved by the board within a year of appointing the team that were recruited to implement it.

**Conclusion:** We propose there is a professional leadership space that psychiatrists have not yet moved to occupy outside of mental health trusts. However, psychiatrists are in fact extremely well placed to contribute to the provision of mental health and learning disabilities in an acute trust given the background of medical and mental health training. The impact of the strategy now needs to be assessed to ensure that it delivers improved services.

